# The Relation of the Chemistry of the Saliva (Sialo-Semeiology) and Nasal Secretions to Diseases of the Mucous Membrane of the Mouth and Upper Respiratory Tract

**Published:** 1904-02

**Authors:** D. Braden Kyle

**Affiliations:** Philadelphia


### THE RELATION OF THE CHEMISTRY OF THE SALIVA (SIALO-SEMEIOLOGY) AND NASAL SECRETIONS TO DISEASES OF THE MUCOUS MEMBRANE OF THE MOUTH AND UPPER RESPIRATORY TRACT.[1]

BY D. BRADEN KYLE, M.D., PHILADELPHIA.

Mr. President and Gentlemen of the Institute of Stomatology,—I know of no subject in which the dental and medical profession should be more intimately interested than the one of *sialo-semeiology.* That the various chemic compounds introduced into or secreted within the mouth have a direct action on the teeth and adjacent structures cannot be doubted. If, then, a secretion has been perverted from the normal, and in this perversion there is eliminated or precipitated some irritating material, such material will not only have a deleterious effect on the teeth, but when taken back into the system will in turn produce some pathologic alteration in other structures.

In the chemic study of the nasal and salivary secretions we may conveniently classify them under three varieties: First, secretions —non-irritating *per se*—which on exposure (when coming to the surface) undergo some chemic change producing an irritant; this may be noted in either an acid or alkaline secretion. As I will

---

[1] Read before The New York Institute of Stomatology, November 6, 1903.

show later, an exceedingly alkaline secretion is decidedly more irritating and productive of a more destructive pathologic process than even a strongly acid secretion. Second, secretions which are irritating *per se* when poured out on the surface. Third, secretions which come to the surface in a non-irritating form, but on coming in contact with extraneous material are rendered irritant. While my observations have been more from a medical stand-point, yet many of the principles involved are applicable in many pathologic lesions as observed by the dental profession.

Two great principles which have been satisfactorily demonstrated are these: In highly alkaline conditions there is an exaggerated oxidation process and the chemic change takes place in the secretion after it is poured out. This is of the greatest importance in nasal, laryngeal, and pharyngeal lesions, and is also of immense significance in pathologic changes in the gums and about the teeth, as highly alkaline secretions will cause necrosis more quickly than an acid secretion. In highly acid conditions the oxidation process is incomplete, hence there is a greater tendency to a precipitation of material within the tissue, with the necessary pathologic alteration—an infiltration process. This as applied to the teeth would produce a class of diseases in which the structure of the tooth is altered through faulty nutrition with a tendency to infiltration, or, in other words, the alkaline condition would produce external alterations going from without in, while the acid condition would produce lesions within with a tendency to come to the surface.

Any one familiar with laboratory investigations involving organic chemistry will appreciate the many hours spent over chemic formulæ and analyses in which the results are negative, through faulty lines of investigation, due to the fact that we are dealing with organic chemistry and searching for unknown quantities. I have purposely omitted from this paper the tedious laboratory details and chemic formulæ, dealing entirely with the practical side of the subject, and I hope at some future date to publish the laboratory formulæ to be used in obtaining reactions of certain pathologic materials which I have found present.

Being so impressed with the import of the study of not only the excretions from the intestines and kidneys, but also of the saliva and various secreting glands, I devoted a number of lectures to this subject during my course on pathology in the Jefferson Medical

College, 1895–96, since carrying on, as time would permit, investigations in this line in my own private laboratory.

Michaels, of Paris, in his admirable paper read before the International Dental Congress, August 9, 1900, was the first, I believe, to call attention to this subject, not in medicine, but in its relation to dentistry; also, Kirk, of Philadelphia, has taken up the investigation.

Naturally, it was necessary to investigate the histo-chemistry of normal saliva, concerning which subject the literature is very meagre. The saliva is the mixed secretion of the parotid, submaxillary, and sublingual glands and the small mucous glands of the mouth. Physiologically, three kinds of secretion may be distinguished,—a serous from the parotid, a mucous from the mucous glands, and a mixed secretion from the submaxillary and sublingual glands. Mixed saliva is opalescent, tasteless, generally alkaline, and has a specific gravity of 1004 to 1009. Saliva contains serum albumin, globulin, mucin, urea, an amylolytic ferment called ptyalin, and a proteolytic and lipolytic ferment; also salts, the most important of which is the ammonium and potassium and the sulphocyanide combinations derived especially from the parotid gland. The proteolytic and lipolytic ferments are not important. It is possible that any other fermentation save the amylolytic is due to bacteria. The amylolytic ferment is most important. The irritating materials produced by this ferment are most significant in causing dental lesions.

The ammonium salts and sulphocyanide in healthy saliva are in equal proportion and in very small quantities; in the hypoacid condition the ammonium salt exists in greater quantities than the sulphocyanide, and tends to rapid decomposition. In the hyperacid condition the sulphocyanide is in excess, and the tendency to decomposition is not so great as in the hypoacid condition until exposed to moisture or air; in other words, after secretion has taken place and chemic action has caused alteration of the compound.

In hyperacid conditions the sulphocyanides are in greater proportion than the ammonium salts, and the secretion is less irritating; while in the hypoacid state the ammonium salts are in excess of the sulphocyanides and the secretion is decidedly irritating, and in many cases in which I have been able to examine the secretions, especially of hay-fever patients, this hypoacid condition has existed.

My studies of the saliva have been very much in the same chemic line as Michaels's. First, the study of the normal healthy saliva; second, the saliva from hypoacid individuals; third, the hyperacid condition. To this I have added the neutral or irregular cases, which are neither normal, hyperacid, or hypoacid.

From my investigations I found that the reaction of the salivary secretions as given by the ordinary litmus test was often faulty and misleading, that owing to chemic changes which had taken place after the secretion is poured out on the surface of the membrane the reaction of the secretion changed. This was of the greatest import from the stand-point of treatment, as the reaction might show precisely the opposite as existed in the secretion as it came from the gland proper.

The condition of normal, hypoacid, and hyperacid conditions can best be illustrated by cases.

(1) Strongly alkaline conditions. The following case illustrates this condition. Dr. B., who had been suffering from an irritating rasping cough since September, 1902, was referred to me in January for an examination of his throat. The symptoms presented were principally the cough, with hyperæmic and irritated mucous membranes, though the congestion and swelling were not in proportion to the severity of the cough. The cough was persistently spasmodic, resembling almost paroxysms of whooping-cough, except more often repeated. There was decided hoarseness and congestion of the vocal cords. The chest examination was negative, with the exception of slight bronchial râles. Here and there on the mucous membrane were small hemorrhagic spots, which I believe had been produced by the violent irritation caused by the spasmodic cough.

In so many cases in which the objective symptoms were in excess of that which the local lesion would justify, I have found that the irritation was brought about by some altered chemistry of the secretion, and that the local lesion was merely a manifestation and result of this alteration. Thus, on examination of his saliva it was found to be hypoacid, the ammonium salts being in excess, and when the secretion came to the surface there was liberated free ammonia gas, which was in sufficient quantity to produce the irritation. On this basis the treatment was administered, all the organs of elimination were stimulated, and the chemistry of the secretion was changed to acid or neutral reaction, following which change the cough disappeared within a few days and the irritation rapidly subsided, and in ten days all inflammation had disappeared with the exception of some slight localized areas, which probably had been brought about by the persistent coughing, and these areas rapidly yielded to local treatment.

To review the chemistry, ammonium is a hypothetical alkaline base, having the composition of $NH_4$; it does occur in a free state, however, in the form of ammonia gas, $NH_3$, the inhalation of which is very irritating to the mucous membrane and causes suffocation and œdema of the glottis. As the ammonium salts usually exist in combination with other materials, is it not likely, in certain conditions in which the secretions are hypoacid, that when the secretion comes to the surface of the mucous membrane, owing to its chemic combination with oxygen, there is liberated $NH_3$, the irritating ammonia gas; surely the symptoms in many cases justify this conclusion, and the chemic study of the secretions supports this view.

Some cases of hay-fever further illustrate this point. The numerous theories as to the etiologic factor in this disease proves conclusively that as yet there has not been established a definite cause. It may be that different conditions act as etiologic factors; in fact, it is my belief that not all cases which we call hay-fever, or hyperæsthetic rhinitis, are due to any one cause; or, if to any one factor, that factor must be in the altered chemistry of the secretions of the individual. In fact, I am persuaded, after making a series of examinations of the saliva in certain individuals afflicted with hay-fever and those not so afflicted, that in some cases the causes, direct or indirect, of local irritation in the nasal mucous membrane are brought about by some chemic change in the constituents of the secretions of the mucus-secreting glands, and that in such cases the reaction of the secretion is strongly alkaline. Treatment based on this view was certainly most effective. Sensitive areas within the nasal cavities, or irregularities in formation of the cavities themselves, are factors in some cases, yet such areas or irregularities, instead of being etiologic, are merely auxiliary factors, which render the individual more susceptible to the irritant from within.

(2) The cases in which the secretions are hyperacid. The following case is an illustration: Mr. C., aged forty-two, consulted me in regard to what he supposed to be a catarrhal condition associated with ozena. His breath was surely most offensive, but, although pronouncedly so, it was not the penetrating, clinging odor observed in atrophic rhinitis with ozena. He had observed the condition rather suddenly, and it had existed continuously for some four or five years. His history was absolutely negative as to any catarrhal condition other than an occasional cold. He had consulted specialists both in this country and abroad, not only as to the possibility of the odor coming from the nose or some of the accessory cavities, but had also consulted specialists on diseases of the stomach, as well as having had a thorough inspection of all his teeth. He had been told that he had practically no catarrh, and as his digestion was good and nothing found wrong by analysis of the contents of the stomach, it was quite puzzling as to the source of this odor. After a thorough examination, and knowing that the men under whose care he had been were most thorough and competent in their line, I reasoned that there must be some source of the disagreeable odor outside of the parts already mentioned. As this was in the winter of 1895, and as my attention had been called to the import of the secretions by other conditions, as well as a statement made to me by the patient, I decided to investigate the saliva. The statement which he made to me, which was most significant, was this: That while his appetite was very good, and when his olfactory nerve was stimulated by the odor of a delicious meal, causing his mouth to water, the disagreeable odor and taste became so pronounced as to almost nauseate him. I collected then some of the saliva. The method I used for its collection I learned from my experience in a dentist's chair: that while sitting with your mouth wide open for a few minutes you have a most profuse flow of saliva. This method, practised just before meal-time, enabled the collection of quite a large amount of the secretion. The offensiveness of the secretion was at once detected.

In order to explain the chemic source of the offensive odor it is necessary to take up the chemistry of the sulphocyanides. As to the sulphocyanides which are present in the saliva, it is a chemic fact that most of the cyanides are actively poisonous and that a cyanide is formed or is a compound of cyanogen with a metal or radical. A sulphocyanide is a salt in which the sulphur takes the place of oxygen in the acid radical. Cyanogen is a radical molecule having the structure CN, an acid compound of carbon and nitrogen. A radical is a group of atoms having unsatisfied valency, an unsatisfied molecule which goes into or out of combination without change to itself and which determines the character of compounds. A sulphocyanide is a combination, then, denoting the chemic combination of sulphur with a radical. When a sulphocyanide is eliminated the secretion in which the ammonium salts are in excess is alkaline, but when it comes in contact with the air, owing to the chemic change which takes place, it becomes an acid radical. Hence in many instances in which from our test we believe the secretion to be acid, it is really hypoacid (alkaline). This is most important, and in many instances in which from the test reaction we are led to conclude the reaction to be acid, it is in reality in the system an alkaline reaction, and only becomes acid when, owing to chemic action due to exposure to air, certain materials are eliminated and the reaction changed. In some of the sulphocyanide combinations in which we get the bad odor, as illustrated in the above case, the chemic change causing such odor is as follows: Sulphur itself is an acid element, which unites with oxygen to form an acid radical, but in a sulpho-salt the sulphur takes the place of the oxygen in the acid radical. The sulphocyanide itself would be a sulpho-salt in which, in combination, the sulphur would take the place of the oxygen and give off sulphuretted hydrogen. In a sulpho-salt the sulphur takes the place of the oxygen in combination to form an acid. A sulphocyanide is a sulpho-salt, and when the sulphocyanide is in excess, when liberated and coming in contact with the secretion which contains moisture ($H_2O$), the sulphur would unite with the hydrogen, giving off free oxygen, and form hydrogen sulphide. A radical is a group of atoms having an unsatisfied valency, and is really unsatisfied molecules which go into or out of combination. It is possible, then, to have in a hyperacid condition many chemic combinations take place. This chemic result surely verified the olfactory diagnosis in this case.

(3) The cases neither hypoacid nor hyperacid. That cell nutrition depends upon the chemistry of its supply is illustrated in disease processes associated with any form of infection or rise of temperature. This opens up an enormous field for speculation and investigation. The amount of infection, the peculiar chemic change produced by temperature, the materials absorbed into the body from infective processes, or the autoinfection from the intestinal tract, would in each condition produce its own peculiar chemic compound. Yet I believe a general basis or standard can be reached, at least sufficiently accurate, from which to draw chemic and clinical deductions. For example, of peculiar effect on various structures in the body, brought about by an altered chemistry, in which the secretions may be neutral or irregular, I will quote from an article which was published in *American Medicine,* February 8, 1902, in which I reported a number of cases of enlargement of the thyroid gland in which the cellular elements of the thyroid structure were increased, the enlargement not being due to distended vessels, cystic condition of the gland, or new growth. I reasoned the matter out as follows: It is a well-known physiologic and therapeutic fact that certain drugs have a selective action on certain tissues or organs of the body; *e.g.,* belladonna, with its selective action on the pharyngeal surface; sodium phosphate, with its selective action on the liver, etc. It is also a physiologic fact that the normal chemistry of the body controls the normal secretions from the various secretory organs, that any perversion from the normal necessarily alters the character and chemistry of the secretion, and that the products of such alteration act as irritants to certain parts of the body; the difference between this and drugs administered is that one is introduced into the body and one is manufactured within the body. I therefore reasoned that under certain conditions there is precipitated—due to perverted chemic reaction—a certain material which, circulating through the blood, had a selective action on certain tissues; in the cases observed such selective action occurred in the thyroid gland, acting as an irritant to that gland. While the treatment of these cases reported was somewhat theoretical, I believe, however, that the drug introduced into the body, by its chemic action, altered the chemistry of the material which was acting as an irritant, either rendering that irritating material inert or forming a compound which was non-irritating.

In regard to the pathologic chemic process producing such reactions of the secretion, it is a well-known clinical and laboratory fact that a study of the products of the secreting organs, which in their excretory functions throw off waste material, gives us by deduction a fair idea of what process is going on within the body. Yet this excretory secretion or material is altered in its chemic composition and controlled by the chemic constituents within the body proper.

There is no question that under certain conditions,—for example, when the secretions are acid or alkaline,—the chemic process taking place within the various secretory glands must vary, and the product of such variation in these unknown quantities must be somewhat the same as the variations we would obtain in dealing in the laboratory with known compounds. In other words, that the body is largely a chemic laboratory, having on hand a certain amount of material and having added to it daily ingredients through the respiratory and alimentary tract. Now, any perverted condition from what is known as the normal chemistry may bring about a series of changes and produce chemic products which may be harmless or productive of disease processes. On no other basis can we explain the various diatheses and the precipitation of certain materials in the tissues of the body.

The altered chemistry of the saliva presents many possibilities from an etiologic stand-point. Many forms of lesions of the mucous membrane of the nose, nasopharynx, larynx, mouth, and gums, as well as diseases of the stomach and intestines, may be brought about by the altered chemistry of the saliva. A great many morbid processes are traced to uric acid in some of its many forms, but I believe that many other substances, especially in hyperacid conditions, equally important are deposited and eliminated, which substances act as irritants, producing apparent local lesions. It is a well-known clinical fact that saliva from certain individuals is exceedingly poisonous, as is indicated by the infectious wounds produced by the bite of such individuals, showing that the saliva may be the site of poisonous pathologic, as well as physiologic, compounds.

Unquestionably the chemic reaction of the secretions of the body is an important factor in the susceptibility of individuals to disease. I think there is no doubt that the fact that at one time the individual resists disease and at another time succumbs can be largely explained on this basis. To be sure, it is a question of resistance on the part of the individual, but that resistance is largely controlled by the chemistry of the cell or secretion. It also demonstrates the fact of the accumulative phenomena of certain of the diseases, as is illustrated in uric acid diathesis, which Haig has described as uric acid storm. There is no reason why these same phenomena could not occur as the result of the accumulation of other materials brought about by chemic changes which lessen oxidation and tend to precipitation and accumulation of various morbid products.

The administration of drugs for the relief of, for example, an infective process probably affects such a process beneficially, owing to the fact that in its action it changes the chemistry of the secretions and blood constituents, thereby producing a chemic compound which either prevents the formation of infectious material or alters the nidus of infection to such an extent that it is not suitable for the growth of bacteria.

While my investigations are incomplete and fragmentary, I am convinced that from the study of the saliva we can determine to a great extent any variation in the chemistry of the body. As these various secreting glands receive from the blood the supply from which they elaborate certain chemic compounds, if an analysis were made of the composition of such secretion it would give a good index to the general condition of the individual, and while in many cases the deductions have to be based on, or rather associated with, clinical observation, I soon found them to be of immense value from a stand-point of diagnosis.

The fact that the reaction of the secretion may be apparently acid when there really is present an alkaline condition explains to us many of the cases in which from an acid basis our treatment has failed. That such a condition may exist has been shown by Douin and Gautrelet in their studies of the blood; that the reaction of the plasma is really acid, and if waste products are not eliminated this acidity is increased. The secretions and excretions then also become of an acid reaction.

The irritating gases which form in the stomach and intestines and produce laryngeal and pharyngeal irritation are the result of chemic changes in the intestinal secretion, and such chemic change in the secretion can be demonstrated by a study of the saliva. That autoinfections and chemic changes in intestinal secretions have a marked general effect on the individual is well known. Such material absorbed into the system will unquestionably alter the chemistry of all the secreting glands, and the compounds formed by such alteration which affect the individual can only be determined by the study of organic chemistry. The asthmatic conditions which are not associated with any organic lesion I believe can all be explained on this basis, and when treated accordingly can in many instances be relieved.

In examining the secretion and excretion of the body, we can obtain as good an index of the systemic condition of the individual by a study of the saliva as by a study of the urine; in the urine we have only an index of the waste material, while in the saliva we have products of elimination which return into the body to perform a physiologic process. Another important factor to be worked out is the different chemic changes which take place in individuals suffering with the so-called functional diseases and those suffering from organic or structural changes. In the functional we have a perverted chemistry, which may be brought about by many causes, such as faulty elimination from the kidneys or liver, and perversion of secretion from the intestinal tract, autoinfection from the intestinal tract; also chemic changes of the secretion illustrated in mental tension, from fright, worry, and anger; while in the organic lesions we have to deal with a structural change in tissues with retrograde metabolic changes, in which there is also associated inflammatory processes with their accompanying phenomena and physiologic and pathologic effect on the individual.

What deductions can be made from the stand-point of the dentist? What effect does the hypoacid condition have on the teeth and alveolar processes? How does it differ from the hyperacid secretions? My own observations have shown that in many cases with highly alkaline secretion there has been irritation of the gums, in some cases amounting to a spongy condition, and in not a few cases pyorrhœa alveolaris, with a tendency to decomposition around the teeth; while in the acid condition the alterations occurred more as a direct lesion of the tooth or nerve, the process beginning from within. This confirms the observations as to the oxidation process. Are these changes due to the altered chemical conditions or to some other cause? I leave it to you, gentlemen, to make the deduction, and, if you find any suggestion worthy of consideration, to apply the same to the pathologic processes occurring within the field of dentistry.

